# Differences Between the 8th and 9th Editions of the TNM Staging System in Predicting Mortality in Non-Small Cell Lung Cancer Patients Staged with EBUS

**DOI:** 10.3390/diagnostics15131570

**Published:** 2025-06-20

**Authors:** Ezgi Demirdöğen, Orkun Eray Terzi, Özge Aydın Güçlü, Ahmet Ursavaş, Mehmet Karadağ

**Affiliations:** 1 Department of Pulmonology, Faculty of Medicine, Bursa Uludağ University, Bursa 16059, Türkiye; ozgeguclu@uludag.edu.tr (Ö.A.G.); ahmetursavas@gmail.com (A.U.); karadag@uludag.edu.tr (M.K.); 2 Department of Pulmonology, University of Health Sciences Bursa Yüksek İhtisas Training and Research Hospital, Bursa 16310, Türkiye; terziorkun@gmail.com

**Keywords:** non-small-cell lung cancer, EBUS, EBUS–TBNA, 9th edition TNM staging, mortality

## Abstract

**Background:** The distinction between N2a and N2b in the lung cancer TNM 9th edition staging system has reduced the heterogeneity of prognosis using the previous staging system. Moreover, this distinction may enable new treatment approaches in non-small-cell lung cancer (NSCLC). We aimed to evaluate the differences in survival between 8th- and 9th-edition staging and the mortality prediction of the TNM 9th edition in NSCLC patients who did not undergo surgical staging and who were “N”-staged with solely endobronchial ultrasound–transbronchial needle aspiration (EBUS–TBNA) without endoscopic ultrasonography (EUS). **Methods:** Lung cancer patients who were newly diagnosed and staged with EBUS between May 2016 and January 2023 were retrospectively reviewed. Patients were divided into two groups, “All M0 = Model 1” and “T1–2 N1–2–3 M0 = Model 2”, and compared according to their survival for both the 8th and 9th edition TNM staging systems. Cox regression analyses were performed for independent predictors of 2-year mortality. **Results:** In this retrospective study, a total of 90 patients were included. Most of the patients were male (84.4%), and the mean age of the study group was 64.0 ± 9.6; deceased patients were older (*p* = 0.024). There were no differences between groups in terms of smoking habit, comorbidities, tumor PET/CT localization, or 8th and 9th N-staging results with EBUS. The median follow-up period was 26 (0–100) months and longer for living patients than deceased patients in both groups (42 (23–100) vs. 18 (0–74), *p* = 0.03; 36 (24–100) vs. 20 (1–74), *p* < 0.001). According to the 8th edition of TNM staging, N2 stage (HR 2.26, 95% CI 1.01–5.05, *p* = 0.045) and N3 disease (HR 3.31, 95% CI 1.43–7.67, *p* = 0.005) are independent predictors of two-year mortality for Model 1 patients. When patients were staged according to the 9th edition TNM with EBUS, the relationship between N2a and mortality was not significant, while N2B disease increased the 2-year mortality risk by 2.78-fold (95% 1.07–7.22, *p* = 0.035), and N3 disease increased it by 3.31-fold (95% 1.43–7.67, *p* = 0.005). **Conclusions:** According to the TNM 9th edition staging system, we demonstrated that N2b disease significantly increases the risk of mortality in NSCLC cases using systematic mediastinal staging with EBUS–TBNA alone.

## 1. Introduction

Lung cancer is the most common cancer worldwide, with almost 2.5 million new cases each year, and it is also the most common cause of cancer-related deaths, with 1.8 million deaths annually [1]. The International Association for the Study of Lung Cancer (IASLC) has established a standardized tumor node metastasis (TNM) staging classification that aims to bring together groups with similar prognoses and treatments based on the anatomic extent of malignancy. The “N” descriptors, categorized by anatomical location, had not been changed from the 4th edition until the recent 9th edition [2,3]. The TNM 8th edition lung cancer staging system has been widely used worldwide since 2017. The “N” descriptor of 8th-edition staging is based on the anatomic location of lymph node metastases, without taking into account the number of metastasized nodes. However, it was emphasized that factors other than the anatomical components of the lymph nodes were also effective in demonstrating prognostic heterogeneity, and it was stated that the lymph nodes should be reclassified into subgroups [4,5,6,7]. These changes in the 9th edition TNM “N” staging resulted from the significant survival difference between N2a and N2b [7]. According to the new staging system, which changed with the subdivision of N2 into N2a and N2b, T1N2a cases were found to have better survival rates than stage IIIA cases according to the 8th TNM edition, similar to stage IIB [8]. These findings led to the assignment of T1N2a tumors to stage IIB. In previous staging approaches, T1N1 tumors classified as stage IIB were downstaged to stage IIA because they had similar survival rates to stage IIA. In contrast, T2 tumors with multiple N2 in stage IIIA were upstaged to IIIB in the 9th edition for similar reasons. T3aN2a cases in the 8th TNM edition, which had been staged as IIIB, were downstaged to IIIA due to similar prognoses [8]. The new “N” descriptor in TNM staging, in addition to being a prognostic factor, plays a critical role in determining suitability for surgery and distinguishing between early-stage and locally advanced disease (N2/N3 status).

In recent guidelines with a high recommendation level, endosonography (EUS/EBUS or EBUS or EUS alone) is recommended as the initial procedure for mediastinal lymph node staging instead of surgical staging in patients with suspected or proven NSCLC with abnormal mediastinal and/or hilar nodes on CT and/or PET [9,10]. In fact, endoscopic staging is recommended even in certain patients with a negative PET [10]. Unlike EUS, EBUS provides unique access to the hilum and structures anterior to the large airways, but EBUS cannot access the lower mediastinal lymph nodes at stations 8 and 9 adjacent to the esophagus, which can be easily targeted with EUS or EUS-B [11]. The “SCORE” study showed that systematic EBUS combined with esophageal ultrasound using the same EBUS bronchoscope (EUS-B) increased the sensitivity of mediastinal lymph node staging in lung cancer patients by 9% compared to targeted EBUS [12]. The combined guidelines of ESGE, ERS, and ESTS recommend that the combination of EBUS/EUS or EBUS/EUS-B be preferred over either procedure alone [10]. In the same guidelines, EBUS alone is considered acceptable in cases in which combined endosonography is not available. In addition, compared to mediastinoscopy, EBUS is an isolated procedure that is associated with lower risks and costs [13]. There are no publications on the effect of “N” staging with EBUS–TBNA without EUS as a mortality predictor in the new TNM edition.

We planned to group the cases with non-metastatic NSCLC diagnoses as deceased and alive in two different models and to compare the demographic features, comorbidities, EBUS–TBNA-verified “N” stages, and PET/CT TNM stages according to both the 8th and 9th editions. We also aimed to evaluate the mortality prediction of the “N” descriptor in the 9th edition staging system in cases in which we performed complete systematic mediastinal staging with EBUS–TBNA without EUS or EUS-B.

## 2. Materials and Methods

### 2.1. Design, Patient Selection, and Data Collection

**Figure 1:**
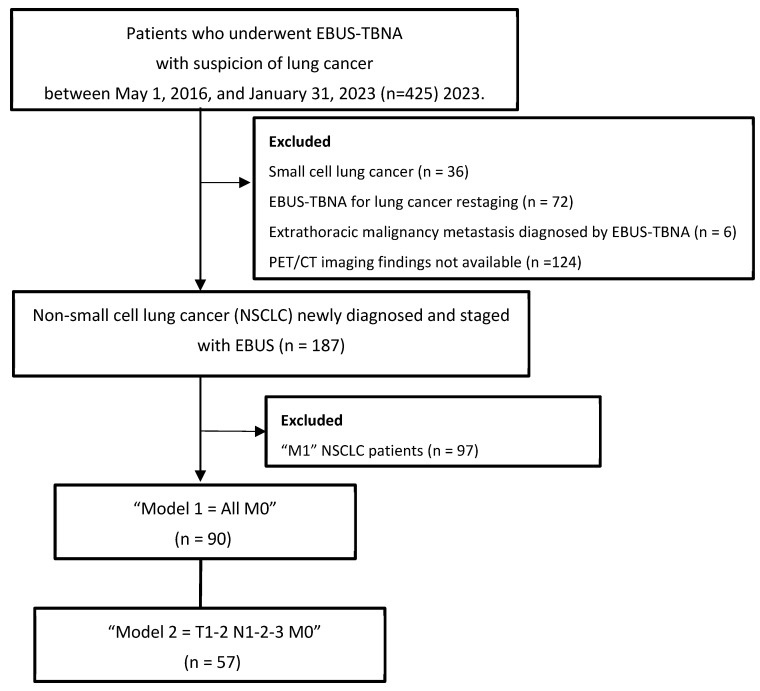
Flowchart of patient selection.

Patients who underwent EBUS for diagnosis and/or mediastinal staging of lung cancer between 1 May 2016 and 31 January 2023 were retrospectively reviewed. Patients with non-small-cell lung cancer (NSCLC) with no metastasis (M0) who underwent complete systematic mediastinal staging with EBUS were included in this study, as shown in the study flowchart (Figure 1). We collected data from medical records, including patient characteristics such as age, smoking history, presenting symptoms, and comorbidities. The duration of follow-up and timing of death were also noted. Tumor location and size, PET/CT findings, and the number of collected lymph nodes with EBUS and EBUS–TBNA pathological results were recorded. For pathological and clinical staging, the location of lymph nodes was categorized based on the IASLC lymph node map [14]. We classified the patients according to the 8th and 9th edition staging systems based on EBUS–TBNA for the “N” stage and PET/CT for the “T” and “M” descriptors. EBUS–TBNA was performed in each patient on all lymph nodes from N3 to N1 that could be reached and sampled, i.e., systematic mediastinal staging. All procedures were performed by one of two experienced bronchoscopists, and samples were processed by the same cytopathologist with no ROSE. EBUS–TBNA samples were air-sprayed into a mixture of 10% formalin and 96% alcohol and then sent to the cytology laboratory for cell block and cytoblock preparation. Air-dried smear preparations were prepared by staining with the May–Grunwald–Giemsa stain in the pathology laboratory.

NSCLC cases with metastasis were excluded from the study population. Patients were examined using two models, “Model 1 = All M0” and “Model 2 = T1–2, N1–2–3, M0”, and compared according to their survival in both the 8th and 9th edition TNM staging systems.

### 2.2. Statistical Analysis

The data were analyzed using IBM SPSS Statistics for Windows, Version 28.0., and *p* < 0.05 was considered statistically significant. The distribution of continuous data was validated using the Shapiro–Wilk test. The continuous data were presented as the mean ± standard deviation (SD), or median (minimum–maximum), while the categorical variables were expressed as *n* (%). For between-group comparisons, parametric independent sample t-tests or non-parametric Mann–Whitney U tests were used depending on the findings of the normality test. Pearson’s chi-square test was performed to compare categorical variables. The multivariate Cox regression model was constructed with the variables that met the *p* < 0.25 threshold in the univariate analysis. The variables were selected using the backward stepwise LR approach, and the findings of the analysis were presented. A summary was provided for the hazard ratios (HRs) and 95% confidence intervals (95% CIs). Kaplan–Meier survival analysis, conducted using MedCalc^®^ Statistical Software version 22.023 (MedCalc Software Ltd., Ostend, Belgium; https://www.medcalc.org; 2024), was employed to assess the impact of the 8th and 9th edition TNM staging systems on overall survival.

An a priori power analysis was conducted using G*Power version 3.1 to determine the required sample size for detecting a significant difference in age between two independent groups. The analysis was based on a one-tailed t-test, with an anticipated effect size (Cohen’s d) of 0.577 (calculated from group means of 65.2 and 60.1 and standard deviations of 10.0 and 7.5, respectively). The alpha error probability was set at 0.05, and the desired statistical power was 0.80. The analysis indicated that a total sample size of 76 participants (38 per group) would be sufficient to detect the expected effect. The resulting noncentrality parameter was 2.52, and the critical t-value was 1.665. The actual power achieved with this configuration was 0.80, confirming the adequacy of the sample size to detect a medium effect.

## 3. Results

Out of the 90 included patients, 76 were male (84.4%), and the mean age of the study group was 64.0 ± 9.6. In the whole group, referred to as “Model 1”, it was seen that the deceased patients were older than the living patients (65.2 ± 10.0 vs. 60.1 ± 7.6, *p* = 0.024, respectively), while in “Model 2”, the age difference between the two groups was not statistically significant (64.8 ± 10.3 vs. 59.3 ± 7.9, *p* = 0.06, respectively). In addition, in “Model 1”, it was seen that the deceased patients had more smoking pack years than the surviving patients [40 (8–144) vs. 33 (10–70), *p* = 0.041, respectively]. No differences were found between deceased and living patients in both models in terms of gender, tumor PET/CT localization, cancer stages by PET/CT, 8th and 9th EBUS “N” stages, and the presence of comorbidities such as hypertension, COPD, diabetes mellitus, coronary artery disease, cerebrovascular disease, asthma, and interstitial lung disease (Table 1). However, in both models, it was observed that subcarinal lymph nodes were sampled significantly more in cases with deceased patients than in cases with surviving patients, as shown in Table 1. The majority of the 90 patients included in this study were at the advanced stage, stage 3 (34 of them were stage 3A, 35 were stage 3B, and 10 were stage 3C). Histopathological subtypes detected in the study population consisting of NSCLC cases were adenocarcinoma (*n* = 52, 57.8%), squamous-cell carcinoma (*n* = 35, 38.9%), and undifferentiated non-small-cell carcinoma (*n* = 3, 3.3%). The distribution of pathological subtypes according to survival was similar in both models (Table 1). No difference was found when comparing patients who died and survived according to the “N” staging confirmed with EBUS using the 8th and 9th editions of the TNM (Table 1). The median follow-up period was 26 (0–100) months in all cases and longer for patients who were alive compared to patients who were deceased in both models, as expected [42 (23–100) vs. 18 (0–74), *p* = 0.03; 36 (24–100) vs. 20 (1–74), *p* < 0.001].

**Figure 2:**
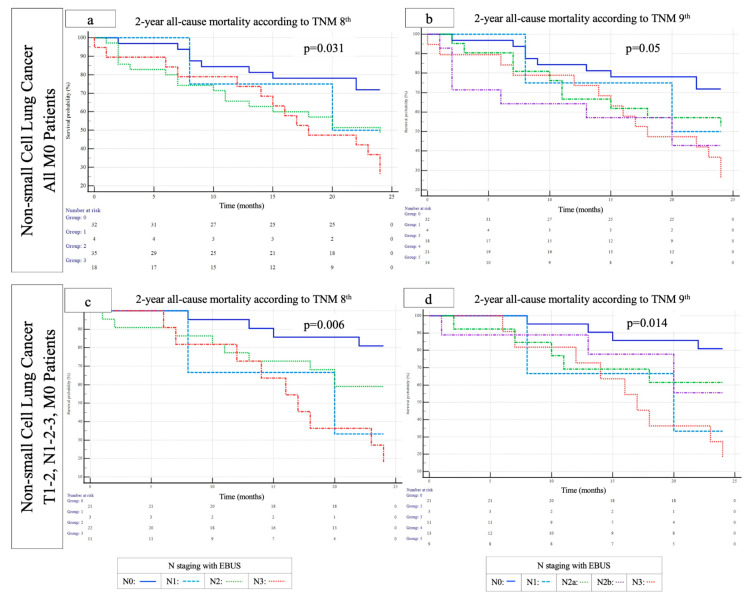
Kaplan–Meier survival analyses for 2-year all-cause mortality in patients with NSCLC according to N staging with EBUS–TBNA based on the 8th and 9th TNM editions. (**a**,**b**) For all M0 patients, mean survival times according to the 8th edition of the TNM classification were 20.6 ± 1.1 months for N0, 19.0 ± 3.2 months for N1, 16.6 ± 1.5 months for N2, and 16.7 ± 1.8 months for N3 (*p* = 0.031), while the corresponding values in the 9th edition TNM classification were 20.5 ± 1.1 months for N0, 19.0 ± 3.2 months for N1, 17.7 ± 1.8 months for N2a, 15.0 ± 12.5 months for N2b, and 16.7 ± 1.8 months for N3 (*p* = 0.05). (**c**,**d**) For patients with T1–2, N1–2–3, M0 disease, mean survival times according to the 8th edition of the TNM classification were 22.1 ± 0.9 months for N0, 17.3 ± 3.9 months for N1, 18.8 ± 1.6 months for N2, and 16.8 ± 2.0 months for N3 (*p* = 0.006), while the corresponding values in the 9th edition TNM classification were 22.1 ± 0.9 months for N0, 17.3 ± 3.9 months for N1, 18.4 ± 2.1 months for N2a, 19.3 ± 2.4 months for N2b, and 16.8 ± 2.0 months for N3 (*p* = 0.014).

In the multivariate Cox proportional hazards regression model including age, smoking habit, and N staging with EBUS, according to the eighth edition of TNM staging, N2 stage (HR 2.26, 95% CI 1.01–5.05, *p* = 0.045) and N3 disease (HR 3.31, 95% CI 1.43–7.67, *p* = 0.005) were independent predictors of 2-year mortality for all M0 patients (Model 1) (Table 2). When patients were staged according to the 9th edition TNM system with EBUS, in the same Cox analysis in non-metastatic patients (Model 1), no significant association was found between N2a and mortality, while N2b disease increased the 2-year mortality risk by 2.78 times (95% 1.07–7.22, *p* = 0.035) and N3 disease by 3.31 times (95% 1.43–7.67, *p* = 0.005) (Table 2). The Kaplan–Meier survival curves for 2-year mortality according to N stage in the previous (8th) and most recent TNM editions (9th) are shown in Figure 2.

## 4. Discussion

In our study, according to the 8th edition TNM staging system, cases of non-metastatic N2 and N3 disease confirmed with EBUS–TBNA were found to be independent predictors of mortality. In “N” staging confirmed with EBUS according to the 9th TNM edition, the N2a stage did not predict mortality, while N2b disease was shown to be an independent predictor of 2-year mortality, with a 2.78-fold increased risk in all M0 patients. However, the same prediction regarding N2b disease was not found in T1–2 N1–3 patients with no metastasis. EBUS-verified N3 disease, when evaluated according to both the 8th and 9th editions, increases the risk of mortality by 3.31 times and 6.37 times in Model 1 and Model 2, respectively.

In a systematic review, the overall mortality rate for lung cancer was found to be 10% (95% CI of 6–16%) [15]. Advanced age, male gender, stage, and the presence of comorbidities such as hypertension, cardiovascular disease, and diabetes mellitus have been shown to have a positive effect on mortality in lung cancer [15,16]. In our study group, no difference was observed in terms of comorbidities in the deceased and surviving groups in both models, and gender distribution and cancer stages according to PET/CT (for both the 8th and 9th editions) were similar. This provided a more homogeneous population for analysis in determining the risk factors affecting mortality. Long-term survival in NSCLC varies significantly according to the endoscopic “N” stage of the disease. In a study examining the prognostic impact of endoscopic N staging in over 1000 NSCLC cases, although patients with endoscopic N0–1/pathological N2–3 disease had worse survival rates than patients with pathological N0 disease, no significant difference was observed compared with patients with pathological N1 disease [17]. Additionally, EBUS has been shown to significantly increase the surgery rate in NSCLC patients with stage II–N1 disease, presumably by reducing the number of patients previously excluded as surgical candidates using PET scans [18]. In the first multicenter randomized controlled trial investigating the added value of mediastinoscopy after negative EBUS results (MEDIASTrial), the unforeseen N2 disease rate was 8.8% in those who underwent immediate resection, which was not lower than the rate of 7.7% in the mediastinoscopy group. As revealed in previous meta-analyses, the results of this study showed that EBUS is not inferior to mediastinoscopy even when determining early-stage cases that are candidates for surgery, and that it is preferable because of its time-saving nature and lower complication rates [19,20,21].

In patients with early-stage NSCLC, systematic mediastinal staging with EBUS–TBNA is known to increase the accuracy of staging. In a meta-analysis, the mean negative predictive value of EBUS–TBNA in detecting radiologically occult, unsuspected N2/N3 metastases was 91% (82–100%) [22]. However, although only targeted lymph node sampling is usually performed in patients with locally advanced NSCLC, even in these cases, systemic mediastinal staging has been shown to be superior to PET in determining the radiotherapy field. In the SEISMIC trial, PET-occult lymph node metastases were detected in 12% of patients following systematic endoscopic staging [23]. However, in patients with NSCLC who had an abnormal mediastinum detected with CT or PET/CT, even if systematic mediastinal staging is not performed, the complete evaluation of all lymph node stations and sampling from at least three different mediastinal lymph node stations, such as both lower paratracheal and subcarinal lymph nodes, are among the ESTS recommendations [10]. In our study, all accessible lymph nodes, not just those that were marked as suspicious using CT and/or PET imaging, were sampled with EBUS, as recommended in the lung cancer staging guidelines [8,10,24]. Complete systematic mediastinal lymph node staging, rather than targeted staging, was especially important for detecting multiple N2 disease, which is one of the significant changes in the new 9th edition of TNM staging. Changes to the TNM 9th edition classification of lung cancer were considered necessary because of the profound difference in survival between N2a and N2b. Analysis has shown that N2a and N2b clearly define prognostically different tumor groups, with data indicating a clear and consistent distinction between single and multiple N2 nodal metastases [25]. One study compared the ability of the 8th and 9th editions to discriminate overall survival and recurrence-free time in over 4000 patients with stage I–III NSCLC who underwent complete curative surgery and concurrent hilar/mediastinal lymphadenectomy [26]. In the aforementioned validation study, the survival difference between N1 and N2a was not as significant as the survival difference between N2a and N2b, suggesting that the number of lymph node metastases has a significant effect on prognosis, as shown in previous studies [27,28].

The guidelines prioritize the combination of EBUS/EUS for the mediastinal staging of NSCLC patients and emphasize the importance of combined endosonography to confirm multiple N2 disease in the new staging system. However, EUS–FNA could not be performed on the patients because it was not available in our center during the study period. In addition, EUS-B also was not performed due to the training requirement for EUS-B and the recent resolution of professional disagreements with gastroenterologists. Only EBUS–TBNA was performed for mediastinal staging in the included cases during the study period. We believe that it is important to demonstrate that EBUS–TBNA alone can maintain mortality prediction, even according to the new staging, when conditions are not suitable for combined endosonography. A meta-analysis has shown that adding EBUS to EUS increases sensitivity by 22%, but the increase in sensitivity is less when EUS is added to EBUS (12%) [29]. Therefore, if both endoscopic methods are not available, as was the case in our center in the recent past, EBUS–TBNA is recommended [10]. Furthermore, EBUS, which provides access to the hilar lymph nodes, should preferably be used if a single endoscopic method is available due to the existence of studies indicating the importance of the presence of N1 accompanying a single N2, which may also cause revisions to staging in the future [30]. In the aforementioned study, the prognosis for single N2 with N1 involvement was found to be worse than the prognosis for single N2 without N1. We found that N2b disease was an independent predictor of 2-year mortality, with a 2.78-fold increased risk in all M0 patients according to systematic 9th-edition TNM “N” staging performed with EBUS alone, without surgical staging or even EUS. In Model 1, which included all cases without metastasis and consisted of 90 patients, there were 33 patients with a more advanced T stage compared to Model 2. However, no 2-year mortality predictions for N2b were observed in Model 2, where T3–4 cases were not included. The reasons for this result may be that the number of cases was insufficient for analysis and that 10 patients who died were Stage 3C patients who were not included in Model 2. In addition, different tumor biology may have been a potential confounder for this result.

We would like to state that during our study, only molecular targeted therapy and immunotherapy are reimbursed for metastatic patients in our country. For this reason, no patient received targeted therapy or immunotherapy in our study, in which we included only non-metastatic cases. It was observed that there was no difference between the deceased and surviving groups in terms of “N” stages detected with EBUS according to 8th- and 9th-edition staging. For this reason, there may not be a significant difference in terms of treatments, especially since we compared relatively homogeneous groups in both models. Excluding metastatic cases and performing mortality analyses in this model makes our results noteworthy. However, difficulties in accessing medical records and not including treatment modalities due to missing data are among the most important limitations of our study. In addition, unlike the previous edition, the 9th edition study also included survival analyses of patients who underwent resection after induction therapy [8]. This situation led to an inconsistency in the survival analyses of patients who did not receive induction therapy. Therefore, the need for a revision with specific survival data for patients receiving induction therapy has been stated in another article by the corresponding author of the project study [31]. In addition to its prognostic significance, “N” staging is also important in deciding suitability for surgery and determining the extent of the disease. However, a change in classification does not automatically mean a therapeutic change, and it is important that treatment decisions are made based on evidence through well-designed clinical studies, as underlined in [31]. Other limitations of our study include its single-center and retrospective design.

## 5. Conclusions

This study showed that the importance of systematic mediastinal staging has increased following the new 9th edition TNM NSCLC staging system, and targeted staging should not be preferred for the detection of multiple N2 disease. Staging with EBUS–TBNA, without EUS, still maintains its place in NSCLC. However, it should not be forgotten that although staging with EBUS–TBNA alone maintains its importance, combined endosonography is the priority recommendation for staging, especially for the confirmation of multiple N2. Moreover, and most importantly, this is the first study to show that multiple N2 disease detected and confirmed with EBUS–TBNA alone, without the addition of EUS or EUS-B, is a significant predictor of mortality.

## Figures and Tables

**Table 1:** Comparison of living and deceased patients’ characteristics, cancer staging results, and factors contributing to overall mortality in two different non-metastatic NSCLC patient groups.

	All M0 NSCLC Patients (*n* = 90)	Model 1 = All M0 NSCLC Patients (*n* = 90)			Model 2 = T1–2 N1–2–3 M0 NSCLC Patients (*n* = 57)		
		Deceased (*n* = 65)	Alive (*n* = 25)	*p*-Value	Deceased (*n* = 40)	Alive (*n* = 17)	*p*-Value
**Age, years**	64.0 ± 9.6	65.2 ± 10.0	60.1 ± 7.6	0.024	64.8 ± 10.3	59.3 ± 7.9	0.06
**Gender, male, *n* (%)**	76 (84.4)	56 (86.2)	20 (80)	0.522	32 (80)	13 (76.5)	0.737
**Smoking habits**							
*Current smoker*	49 (54.4)	36 (55.4)	13 (52.0)	0.949	20 (50)	9 (52.9)	0.912
*Ex-smoker*	28 (31.1)	20 (30.8)	8 (32.0)		14 (35)	5 (29.4)	
*Never smoked*	13 (14.4)	9 (13.8)	4 (16.0)		6 (15)	3 (17.6)	
**Smoking history, pack-year med (min–max)**	40 (8–144)	40 (8–144)	33 (10–70)	0.041	40 (8–144)	40 (15–70)	0.689
**Presence of comorbidities, *n* (%)**	81 (90)	61 (93.8)	20 (80)	0.109	27 (67.5)	13 (76.5)	0.752
**Comorbidity, *n* (%)**							
*Hypertension*	30 (33.3)	21 (32.3)	9 (36.0)	0.805	13 (32.5)	7 (41.2)	0.557
*COPD*	22 (24.4)	18 (27.7)	4 (16.0)	0.288	10 (25)	3 (17.6)	0.734
*Diabetes mellitus*	17 (18.9)	13 (20.0)	4 (16.0)	0.771	7 (17.5)	4 (23.5)	0.598
*Coronary artery disease*	15 (16.7)	12 (18.5)	3 (12)	0.545	5 (12.5)	2 (11.8)	0.938
*Extrathoracic malignancy*	6 (6.7)	5 (7.7)	1 (4.0)	0.529	4 (10)	1 (5.9)	0.615
*Cerebrovascular disease*	3 (3.3)	3 (4.6)	0	0.557	2 (5)	0	0.348
*Asthma*	4 (4.4)	4 (6.2)	0	0.573	3 (7.5)	0	0.542
*Interstitial lung disease*	2 (2.2)	1 (1.5)	1 (4)	0.481	1 (2.5)	1 (5.9)	0.511
*Congestive heart disease*	1 (1.1)	1 (1.5)	0	0.533	1 (2.5)	0	0.511
*Arrhythmia*	1 (1.1)	1 (1.5)	0	0.533	1 (2.5)	0	0.511
*Chronic renal failure*	1 (1.1)	1 (1.5)	0	0.533	1 (2.5)	0	0.511
*Autoimmune disease*	1 (1.1)	1 (1.5)	0	0.533	1 (2.5)	0	0.511
**Tuberculosis history**	5 (5.6)	4 (6.2)	1 (4.0)	0.689	3 (7.5)	0	0.547
**Lung cancer pathological classification *n* (%)**							
*Adenocarcinoma*	52 (57.8)	36 (55.4)	16 (64.0)		27 (67.5)	10 (58.8)	
*Squamous-cell carcinoma*	35 (38.9)	28 (43.1)	7 (28.0)	0.170	12 (30)	5 (29.4)	0.353
*Undifferentiated non-small-cell carcinoma*	3 (3.3)	1 (1.5)	2 (8.0)		1 (2.5)	2 (11.8)	
**PET/CT tumor localization**							
*Right upper lobe*	32 (35.6)	26 (40.0)	6 (24.0)	0.295	18 (45)	4 (23.5)	0.112
*Right middle lobe*	14 (15.6)	7 (10.8)	7 (28.0)		2 (5)	4 (23.5)	
*Right lower lobe*	10 (11.1)	7 (10.8)	3 (12.0)		5 (12.5)	3 (17.6)	
*Left upper lobe*	29 (32.2)	21 (32.3)	8 (32.0)		11 (27.5)	6 (35.3)	
*Left lower lobe*	5 (5.6)	4 (6.2)	1 (4.0)		4 (10)	0	
*Mass long axis (mm) med (min–max)*	30 (12–90)	30 (12–90)	30 (15–70)	0.220	25 (13–48)	23 (15–44)	0.426
*Mass short axis (mm) med (min–max)*	25 (10–73)	25 (10–73)	25 (12–60)	0.151	23 (10–42)	20 (12–40)	0.238
*Mass SUV med (min–max)*	10.0 (1.81–38.88)	10 (1.81–38.88)	10.9 (3.60–36.30)	0.405	9.80 (3.40–38.88)	7.68 (3.60–15.0)	0.005
**PET staging, 8th TNM edition**							
*1A*	1 (1.1)	1 (1.5)	0	0.172	1 (2.5)	0	0.684
*1B*	4 (4.4)	3 (4.6)	1 (4.0)		2 (5)	1 (5.9)	
*2B*	6 (6.7)	5 (7.7)	1 (4.0)		4 (10)	1 (5.9)	
*3A*	34 (37.8)	20 (30.8)	14 (56.0)		18 (45)	11 (64.7)	
*3B*	35 (38.9)	26 (40.0)	9 (36.0)		15 (37.5)	4 (23.5)	
*3C*	10 (11.1)	10 (15.4)	0		-	-	
**PET staging, 9th TNM edition**							
*1A*	1 (1.1)	1 (1.5)	0	0.110	1 (2.5)	0	0.344
*1B*	4 (4.4)	3 (4.6)	1 (4.0)		2 (5)	1 (5.9)	
*2A*	4 (4.4)	4 (6.2)	0		4 (10)	0	
*2B*	12 (13.3)	6 (9.2)	6 (24.0)		5 (12.5)	6 (35.3)	
*3A*	23 (25.6)	14 (21.5)	9 (36.0)		10 (25)	4 (23.5)	
*3B*	36 (40.0)	27 (41.5)	9 (36.0)		18 (45)	6 (35.3)	
*3C*	10 (11.1)	10 (15.4)	0		-	-	
**Number of malignant lymph nodes sampled with EBUS**							
*2R (n = 3)*	2 (66.7)	1 (100)	1 (50)	0.386	1 (100)	1 (50)	0.386
*4R (n = 66)*	32 (48.5)	24 (50.0)	8 (44.4)	0.911	13 (44.8)	6 (50)	0.926
*4L (n = 35)*	17 (48.6)	15 (55.6)	2 (25.0)	0.316	10 (55.6)	2 (33.3)	0.315
*7 (n = 82)*	29 (35.4)	23 (39.7)	6 (25.0)	0.006	16 (44.4)	4 (25)	0.021
*10R (n = 34)*	10 (29.4)	9 (39.1)	1 (9.1)	0.146	7 (43.8)	1 (14.3)	0.373
*11R (n = 49)*	15 (30.6)	9 (27.3)	6 (37.5)	0.752	3 (13)	4 (36.4)	0.208
*11L (n = 62)*	14 (22.6)	12 (26.7)	2 (11.8)	0.228	7 (24.1)	2 (15.4)	0.570
**Eighth TNM edition “N” stage, with EBUS**							
*N0*	32 (35.6)	20 (30.8)	12 (48.0)	0.398	13 (32.5)	8 (47.1)	0.686
*N1*	4 (4.4)	3 (4.6)	1 (4.0)		2 (5)	1 (5.9)	
*N2*	35 (38.9)	26 (40.0)	9 (36.0)		16 (40)	6 (35.3)	
*N3*	19 (21.1)	16 (24.6)	3 (12.0)		9 (22.5)	2 (11.8)	
**Ninth TNM edition “N” stage, with EBUS**							
*N0*	32 (35.6)	20 (30.8)	12 (48.0)	0.549	13 (32.5)	8 (47.1)	0.781
*N1*	4 (4.4)	3 (4.6)	1 (4.0)		2 (5)	1 (5.9)	
*N2a*	21 (21.0)	16 (24.6)	5 (20.0)		10 (25.0)	3 (17.6)	
*N2b*	14 (15.6)	10 (15.4)	4 (16.0)		6 (15.0)	3 (17.6)	
*N3*	19 (21.1)	16 (24.6)	3 (12.0)		9 (22.5)	2 (11.8)	
**Follow-up period, months**	26 (0–100)	18 (0–74)	42 (23–100)	0.003	20 (1–74)	36 (24–100)	<0.001

Data are presented as means ± SD, median (min–max), and *n* (%).

**Table 2:** Independent variables affecting 2-year overall mortality in two models according to the 8th and 9th TNM editions.

	Model 1 NSCLC, All M0 Patients			Model 2 NSCLC, T1–2 N1–2–3 M0 Patients		
	HR	95% CI	*p*-Value	HR	95% CI	*p*-Value
**N staging with EBUS according to the 8th edition of the TNM**						
*(Ref. N0)*						
N1	1.94	0.42–9.01	0.395	4.65	0.85–25.50	0.076
N2	2.26	1.01–5.05	0.045	2.59	0.79–8.41	0.113
N3	3.31	1.43–7.67	0.005	6.37	1.94–20.83	0.002
**N staging with EBUS according to the 9th edition of the TNM**						
*(Ref. N0)*						
N1	1.94	0.42–9.01	0.394	4.65	0.85–25.50	0.076
N2a	1.97	0.80–4.86	0.139	2.51	0.67–9.38	0.169
N2b	2.78	1.07–7.22	0.035	2.67	0.67–10.74	0.163
N3	3.31	1.43–7.67	0.005	6.37	1.94–20.83	0.002

## Data Availability

The original contributions presented in this study are included in the article. Further inquiries can be directed to the corresponding author.

## Abbreviations

The following alphabetically listed abbreviations are used in this article:

EBUS	Endobronchial ultrasound
EBUS–TBNA	Endobronchial ultrasound–transbronchial needle aspiration
ERS	European Respiratory Society
ESGE	European Society of Gastrointestinal Endoscopy
ESTS	European Society of Thoracic Surgery
EUS	Endoscopic (esophageal) ultrasound using a gastrointestinal scope
EUS-B	Endoscopic (esophageal) ultrasound using an EBUS scope
EUS-FNA	Endoscopic (esophageal) ultrasound with fine needle aspiration, with the use of a gastrointestinal scope
HR	Hazard ratio
NSCLC	Non-small-cell lung cancer
PET/CT	Positron emission tomography/computed tomography
ROSE	Rapid on-site evaluation
TNM	Tumor-node-metastasis
